# Direct Repair of the Crystal Structure and Coating Surface of Spent LiFePO_4_ Materials Enables Superfast Li-Ion Migration

**DOI:** 10.1007/s40820-025-01980-1

**Published:** 2026-01-05

**Authors:** Yuanqi Lan, Jianfeng Wen, Yatian Zhang, Xuexia Lan, Tianyi Song, Jie Zhu, Jing Peng, Wenjiao Yao, Yongbing Tang, Hui-Ming Cheng

**Affiliations:** 1 Advanced Energy Storage Technology Research Center, Shenzhen Institutes of Advanced Technology, Chinese Academy of Sciences, Shenzhen, 518055 People’s Republic of China; 2 University of Chinese Academy of Sciences, Beijing, 100049 People’s Republic of China; 3 Shenzhen Key Laboratory of Energy Materials for Carbon Neutrality, Shenzhen, 518055 People’s Republic of China

**Keywords:** Spent Li-ion battery, LiFePO_4_ cathode, Direct recycling

## Abstract

The rapid accumulation of spent LiFePO_4_ (LFP) cathodes from retired lithium-ion batteries necessitates the development of effective and environmental-friendly recycling strategies. In this context, direct regeneration has emerged as a promising approach for reclaiming LFP cathode materials, offering a streamlined pathway to restore their electrochemical functionality. We report an integrated regeneration protocol that simultaneously repairs the degraded crystal structure and reconstructs the damaged carbon coating in spent LFP. The regenerated cathode material had superfast lithium-ion diffusion kinetics and a stable cathode–electrolyte interface, giving a remarkable rate capability with specific capacities of 122 mAh g^−1^ at 5C and 106 mAh g^−1^ at 10C (1C = 170 mA g^−1^). It also maintained capacities of 110.7 mAh g^−1^ (5C) and 84.1 mAh g^−1^ (10C) after 400 cycles. It could be used in harsh environments and could be stably cycled at subzero temperatures (− 10 and − 20 °C) and in solid-state electrolyte batteries. Life cycle assessment combined with economic evaluation using the EverBatt model reveals that this direct regeneration approach has high economic and environmental benefits.

**Supplementary Information:**

The online version contains supplementary material available at 10.1007/s40820-025-01980-1.

## Highlights

- Simultaneously repairing the degraded crystal structure and reconstructing the damaged carbon coating in spent LiFePO_4_ cathode enables superfast lithium-ion diffusion kinetics and produces a stable cathode–electrolyte interface.
- The regenerated LiFePO_4_ cathode delivers remarkable rate capability, low-temperature performance and compatibility in solid-state batteries.
- The proposed direct regeneration approach has high economic and environmental benefits compared to hydrometallurgical and conventional direct recycling methods.

**Supplementary Information:**

The online version contains supplementary material available at 10.1007/s40820-025-01980-1.

## Graphical abstract

**Figure:**
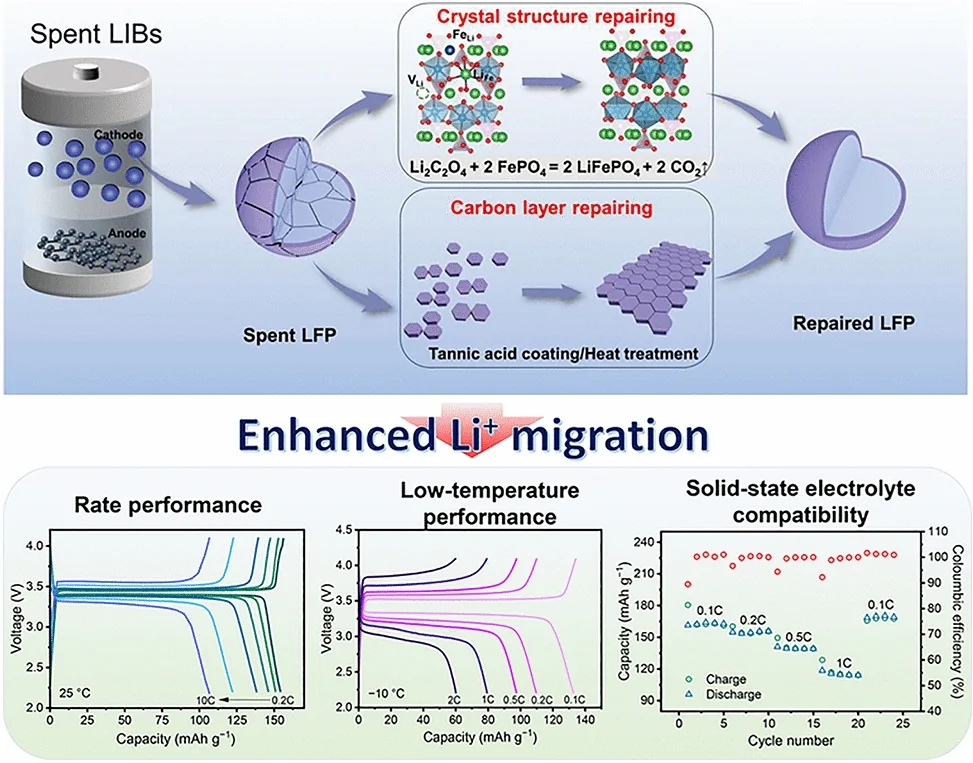

## Introduction

Lithium-ion batteries (LIBs) have become dominant power source for electric vehicles (EVs) and grid-scale energy storage systems [1–4], especially those using LiFePO_4_ (LFP) as cathodes [5–7]. With first-generation EV batteries approaching their end of life, there is a critical need to develop sustainable solutions for LFP recycling [8–11]. Conventional pyrometallurgical and hydrometallurgical recycling processes are not cost-effective because of their large energy demands and high reagent consumption [12]. Furthermore, the inherent volatility in the price of the lithium salt increases the financial risk associated with these conventional methodologies that mainly focus on the recovery of lithium compounds [13]. As a result, direct regeneration strategies that restore the structural integrity of degraded cathode materials using targeted crystal repair rather than complete material decomposition, have received increasing interest.

The key failure mechanisms of LFP cathode materials are generally regarded as a damaged crystal structure, including Li loss, Li-Fe anti-site defects (Fe_Li_), irreversible phase transitions and a damaged surface coating layer [14–16]. Recent advances in direct regeneration have demonstrated that the structural recovery of spent LFP (s-LFP) has three essential requirements: (i) a reductive environment to reverse Fe oxidation states, typically achieved through organic reductants like ethanol [17], glycerol [18], lithium triethyl borohydride [19], polycyclic aromatic hydrocarbons [20] or citric acid [21], (ii) lithium supplementation using Li salts such as LiNO_3_ [22], Li_2_CO_3_ [16, 23] or LiOH [21, 24–26] and (iii) coating regeneration to form a uniform carbon layer using glucose, polyvinylidene fluoride, etc. [27, 28]. While pioneering work by Ji et al. used 3,4-dihydroxybenzonitrile dilithium as a multifunctional regeneration agent achieving 88% capacity retention after 400 cycles at 5C [29], its commercial viability is limited by toxicity concerns and prohibitive costs. Meanwhile, most hydrothermal-related reports use multiple reagents and over 140 °C reactions [30, 31]. This highlights the urgent need for developing benign, cost-effective regeneration protocols that remove both structural defects and surface damage.

We report an integrated regeneration strategy that combines low-temperature hydrothermal relithiation with surface engineering. Our approach uses lithium oxalate (Li_2_C_2_O_4_) to simultaneously provide lithium replenishment and produce reductive conditions at sub-100 °C temperatures. Subsequent surface modification using tannic acid (TA) self-polymerization and thermal annealing achieves comprehensive reconstruction of both the olivine crystal structure and a uniform conductive carbon coating layer. The regenerated LFP cathode material has superfast Li^+^ migration kinetics that contribute to an exceptional rate capability (122 mAh g⁻1 at 5C, 107 mAh g⁻1 at 10C), remarkable low-temperature performance (97.2% capacity retention after 200 cycles at − 10 °C) and excellent compatibility in solid-state battery configurations. Techno-economic analysis confirms the process is favorable for both the economy and the environment, and provides an integrated, effective, economical and environmental-friendly strategy to achieve the direct regeneration of the spent LFP by repairing both the crystal structure and the carbon layer, to give an excellent performance. This work offers new insights for sustainable battery recycling technologies.

## Experimental Section

### Materials

Reagents including Li_2_C_2_O_4_, TA, NaOH, N-methyl-2-pyrrolidone (NMP), poly(vinylidene fluoride-co-hexafluoropropylene) (PVDF-HFP, Mw ~ 400,000), lithium bis (trifluoromethanesulfonyl)imide (LiTFSI, 99.9%) and N, N-dimethylformamide (DMF, 99.9%) were purchased from Aladdin (Shanghai, China). Coin cells, Celgard glass-fiber membranes, polyvinylidene fluoride (PVDF), carbon black, Super P, aluminum (Al) foil, copper (Cu) foil and Li metal disks (16 mm) were purchased from Guangdong Canrd New Energy Technology Co., Ltd. LiPF_6_, ethylene carbonate (EC), ethyl methyl carbonate (EMC) and the electrolyte designed for low-temperature testing (LB-141) were purchased from DoDochem. Commercial LFP (c-LFP) was purchased from BTR New Material Group Co., Ltd. 5 wt% Nafion solution in propanol, and water (D-520) was purchased from Du Pont China Holding Co. Ltd. S-LFP cathode black mass was received form Guangdong Teamgiant New Energy Technology Co., Ltd.

### Preparation of Materials

#### Spent LFP Cathode Materials Pretreatment

The s-LFP cathode black mass was first stirred in the NMP to remove the residual PVDF and then filtered and stirred in a 2-M NaOH solution for 10 h to remove the residual Al foil. Part of carbon is also lost in this process. It was then washed in deionized water, filtered and dried in ovens at 100 °C for 1 day to remove water. The obtained s-LFP was used in following studies.

The delithiated LFP sample was prepared by adding s-LFP to a 50-mL Na_2_S_2_O_8_ solution (s-LFP: Na_2_S_2_O_8_ = 1:0.25, mole ratio) and stirred the mixture for 0.5 h. Then, the resulting products was washed, filtered and collected, dried in an oven at 80 °C for 12 h.

#### Regeneration of LFP Cathode

s-LFP cathode powder (1 g) was added to a 20-mL Teflon-lined autoclave filled with 5 mL water, and Li_2_C_2_O_4_ (1 mmol) was added. The autoclave was heated at different temperatures (180 to 80 °C) and time to check feasibility. The resulting products were washed, filtered, collected and dried in an oven at 80 °C for 12 h. The obtained powder was named LFP-Hydro. 1 g LFP-Hydro was stirred in 10 mL deionized water with 50 mg TA for 15 min. The treated powder was washed, filtrated, collected and dried in an oven at 80 °C for 12 h to produce LFP-Hydro-TA. It was then sintered in Ar flow at 600 °C for 4 h with a heating rate of 5 °C min^−1^. The final processed powder was named as re-LFP. For the regeneration of the delithiated LFP sample, the hydrothermal reaction was repeated until the collected XRD pattern was satisfying.

#### Preparation of the Solid-State Electrolyte

PVDF-HFP, LiTFSI and DMF were used without further treatment. The PVDF-HFP electrolyte was first prepared by dissolving 1.2 g PVDF-HFP and 0.6 g LiTFSI in 5 mL DMF, and then stirred for 12 h to obtain a homogeneous slurry. The slurry was doctor-bladed onto the surface of a glass plate which was then placed in a vacuum oven and dried at 90 °C for 24 h, to obtain the PVDF-HFP electrolyte membrane.

### Coin Cell Assembly

The samples (c-LFP, s-LFP, LFP-Hydro, none TA coated LFP [LFP-Hydro-HT], re-LFP) were mixed with PVDF and carbon black in NMP in mass ratios of 8:1:1, and homogeneous slurry coated on Al foil (17 μm thick) with a 200 μm blade and dried in vacuum at 80 °C for 15 h. The cathode disks with a diameter of 10 mm and mass loading of 2.0–2.5 mg cm^−2^ were obtained on an electrode cutting machine. Coin cells (CR2032) were assembled in an Ar-filled glovebox (O_2_ < 0.1 ppm, H_2_O < 0.1 ppm), with a Li metal disk as the anode, 1 M LiPF_6_ in EC: DMC (1:1 Vol%) as the electrolyte, and a Celgard membrane as the separator.

For the fabrication of solid-state electrolyte batteries, the cathode was prepared by mixing re-LFP, Super P, PVDF, LiTFSI in weight ratios of 75:10:10:5 in NMP, followed by casting the resulting slurry on an Al foil. After drying at 100 °C for 12 h, the cathode was prepared with a mass loading of 1.5–2.0 mg cm^−2^. With the PVDF-HFP electrolyte, lithium anode and LFP cathode, solid-state Li||LFP coin cells were assembled in an Ar-filled glovebox (O_2_ < 0.1 ppm, H_2_O < 0.1 ppm).

### Characterization of Materials

The morphology of the powders was studied by a scanning electron microscope (SEM, Hitachi SU8010, Japan). The crystal structure (ex situ XRD, in situ temperature-varied XRD) of the powders was examined by an X-ray powder diffractometer (Rigaku SmartLab, Japan) using Cu Kα radiation. The XRD refinement was carried out on the GSAS-II platform [32]. The cathode powders were analyzed by X-ray photoelectron spectrometer (XPS, ULVAC-PHI, 5000 VersaProbe II, Japan). The crystal structure and morphology were studied by transmission electron microscope (TEM, FEI, Tecnai F30, USA) and high-resolution transmission electron microscope (HRTEM, FEI, Tecnai F20, USA). Fourier transform infrared spectroscopy (FTIR) spectra of the cathode powders were obtained using a PerkinElmer Frontier instrument (USA). Raman spectra of the cathode powder were obtained using a HORIBA, XploRA PLUS detector in the backscattering mode at the 800–1800 cm^−1^ frequency range, making sure that the intensity of the laser light was low enough to ensure the integrity of the samples. The contents of metal elements in the samples were determined by inductively coupled plasma-optical emission spectrometry (ICP-OES, Agilent-720, USA). Thermal gravimetric analysis (TGA) measurements were performed with a PerkinElmer apparatus. The temperature was increased from RT to 800 °C at the rate of 5 °C min^−1^ in air. Galvanostatic charge–discharge (GCD) tests were carried out using a Neware battery testing system in the potential range 2.2–4.1 V. Electrochemical impedance spectroscopy (EIS), cyclic voltammetry (CV) tests of coin cells were carried out in the fully discharged state using a Donghua electrochemical workstation. Potentiostatic intermittent titration technique (PITT) of coin cell was carried out using a Donghua electrochemical workstation, with a titration potential of 5 mV and relaxation time of 8 h. The voltage was swept from open-circuit voltage (~ 2.8 V) to 4.1 V. The three-electrode CV test was conducted using an electrochemical workstation (Princeton Applied Research). The s-LFP powder was dispersed in a 5% Nafion propanol/water solution. The obtained suspension was dropped and dispersed on a foam nickel electrode. After drying, the electrode was assembled in a cell as the working electrode, and Pt foil and an Ag/AlCl electrode were used as counter electrode and reference electrode, respectively. The electrolyte was the same as hydrothermal solution, i.e., 0.2 mol L^−1^ Li_2_C_2_O_4_.

### Economic and Environmental Analysis

The EverBatt model, a closed-loop battery recycling model developed at Argonne National Laboratory was used to conduct techno-economic and life-cycle analysis of hydrometallurgical, conventional direct regeneration and our proposed recycling processes. The prices of the input and output chemicals used were the current prices in the Chinese market. The specific input of chemicals for the three methods are listed in Table S1.

## Results and Discussion

### Microstructure Characterization of LFP

**Fig. 1:**
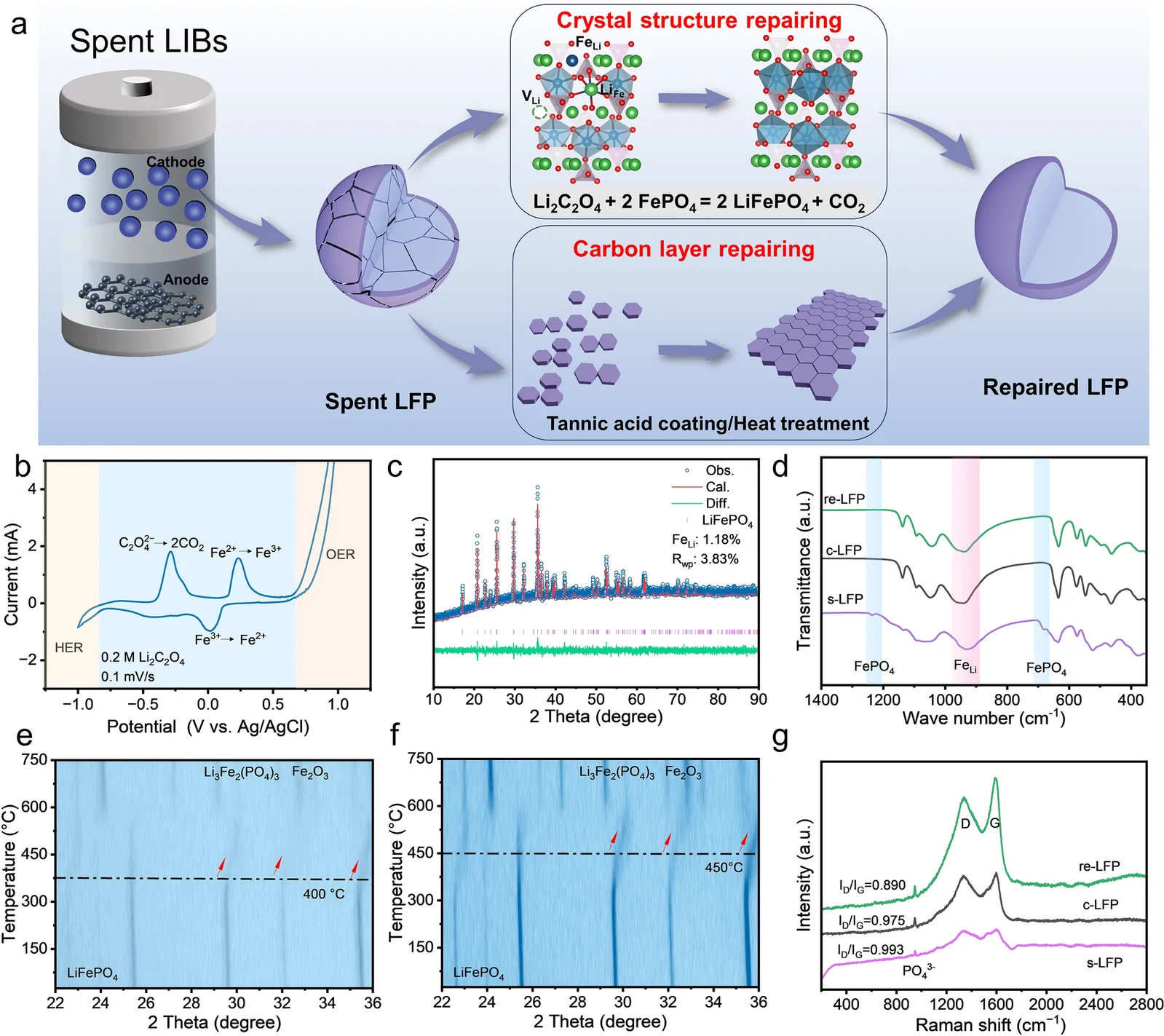
Characterization of LFP samples. **a** Schematic of the direct regeneration of s-LFP in this study. **b** CV curve of the three-electrode configuration. **c** XRD refinement pattern of re-LFP. **d** FTIR patterns of s-LFP, c-LFP and re-LFP. In situ XRD pattern of **e** s-LFP and **f** re-LFP at different temperatures. **g** Raman spectra of s-LFP, re-LFP and c-LFP

The Fe_Li_ and Li vacancies in LFP are the main reasons for sluggish Li-ion diffusion and loss of electrochemical performance [33]. Fe_Li_ defect elimination and relithiation are therefore required to restore the structure. The damage to the carbon coating caused by prolonged electrochemical cycling and battery pretreatment also compromises its stability. To address these problems, we have developed a reconstruction strategy that combines repair of the crystal structure and reconstruction of the carbon layer, as illustrated in Fig. 1a. Li_2_C_2_O_4_ was used in mild hydrothermal conditions at 95–100 °C to produce both reduction of the Fe(III) and filling of the Li vacancies (Eq. 1),

1 $${\mathrm{Li}}_{{2}} {\mathrm{C}}_{{2}} {\mathrm{O}}_{{4}} + {\text{ 2FePO}}_{{4}} \to {\mathrm{2LiFePO}}_{{4}} + {\text{ 2CO}}_{{2}} \uparrow$$

which is shown by the fact that it facilitates Fe(III)-to-Fe(II) conversion and lowers the lithiation energy barrier [17, 20], producing a material that is designated LFP-Hydro. The LFP-Hydro was then stirred in a TA solution to form a uniform TA coating on its surface (LFP-Hydro-TA). TA is selected because of its abundant phenolic hydroxyl groups, excellent antioxidant properties and good solubility in water [34], which enable it to adhere tightly to the LFP surface by Fe-TA chelation. It is also a natural polyphenol that is abundant in plants, which has been used in the leather, coating, adhesion, surgery, pharmaceutical and food industries [33]. The LFP-hydro-TA was then heat treated in Ar at 600 °C for 4 h, and the s-LFP was regenerated, which we refer to re-LFP. The reconstructed carbon coating was visually confirmed by its darker color than s-LFP (Fig. S1).

To demonstrate the feasibility of this method, we used three-electrode CV test to calculate the Gibbs free energy (Δ*G*) of Eq. 1 [21]. The CV curve is shown in Fig. 1b. Oxygen evolution reaction and hydrogen evolution reaction happen when the voltage is above 0.65 V (vs. Ag/AgCl) and below − 0.80 V (vs. Ag/AgCl), respectively. The reaction during CV cycling can be expressed by Eqs. 2–4:

2 $${\mathrm{Reduction}}: {\mathrm{FePO}}_{{4}} + {\text{ Li}}^{ + } + {\text{ e}}^{ - } \to {\mathrm{LiFePO}}_{{4}}$$

3 $${\mathrm{Oxidation}}: {\mathrm{C}}_{{2}} {\mathrm{O}}_{{4}}^{{{2} - }} \to {\mathrm{2CO}}_{{2}} + {\text{ 2e}}^{ - }$$

4 $${\mathrm{Overall}}:{\text{ 2Li}}^{ + } + {\text{ 2FePO}}_{{4}} + {\text{ C}}_{{2}} {\mathrm{O}}_{{4}}^{{{2} - }} \to {\mathrm{2LiFePO}}_{{4}} + {\mathrm{2CO}}_{{2}} \uparrow$$

According to the CV curve, E(FePO_4_/LiFePO_4_) = 0.018 V (vs. Ag/AgCl) and E(C_2_O_4_^2−^/CO_2_) =  − 0.291 (vs. Ag/AgCl). Therefore, the Gibbs free energy of the overall reaction calculated using Eq. 5:

5 $$\Delta G \, = \, - {\text{nFE }} = \, - {\mathrm{nF}}\left( {{\mathrm{E}}\left( {{\mathrm{FePO}}_{{4}} /{\mathrm{LiFePO}}_{{4}} } \right) \, - {\text{ E}}\left( {{\mathrm{C}}_{{2}} {\mathrm{O}}_{{4}}^{{{2} - }} /{\mathrm{CO}}_{{2}} } \right)} \right)$$

to be − 59.6 kJ mol^−1^ at room temperature and pressure, indicating that the relithiaiton of s-LFP by the Li_2_C_2_O_4_ solution is thermodynamically favorable.

The Rietveld refined XRD pattern of s-LFP is shown in Fig. S2. It contains 0.5 wt% FePO_4_ and 99.5 wt% LFP due to Li loss during the previous electrochemical cycling. The Fe_Li_ defect concentration in s-LFP was calculated to be 4.12% (Table S2). Elemental analysis was performed by ICP-OES and showed that there was a lithium deficiency in the s-LFP (Table S3). The optimized hydrothermal reaction condition was determined to be 100 °C for 4 h or at 95 °C for 10 h (Fig. S3 and Table S4), which is favorable considering energy cost and time efficiency. The XRD of re-LFP showed that the Fe_Li_ defect concentration decreased to 1.18%, and no FePO_4_ was detected, as shown in Fig. 1c and Table S5. These results are supported by the FTIR results in Fig. 1d, where c-LFP is also added as a reference. The peak at around 957 cm^−1^ is the symmetric stretching P–O vibration peak of the [PO_4_] tetrahedron [35], and the surrounded Fe_Li_ defects affects the force constant of P and O atoms in the [PO_4_] tetrahedron, resulting in the red shifting behaviors of FTIR spectra [15]. The characteristic feature of Fe_Li_ at around 950 cm^−1^ (red shadow range) in the re-LFP shifts to a higher wavenumber than that of s-LFP (930 cm^−1^), which indicates the lower content of Fe_Li_ defects in re-LFP compared s-LFP. The features at 680 and 1240 cm^−1^ (blue shadow range) in s-LFP disappeared in re-LFP, indicating the FePO_4_-to-LFP phase transformation. In addition, Fig. S4 shows that these characteristic features of re-LFP are consistent to those of LFP-hydro and LFP-Hydro-TA, indicating the Fe_Li_ defects eliminating and relithiation are accomplished in the hydrothermal process.

To check the feasibility of the proposed regeneration method on the more degraded LFP, the as-obtained s-LFP was further delithiated and then gone through the regeneration process repeatedly. Each sample was checked by XRD, as shown in Fig. S5. It can be seen that the delithiated sample contains about 45.5 wt% FePO_4_. After repeatly hydrothermal reactions, LFP phase ratio progressively increased. The sample was fully recovered upon four-time hydrothermal reactions. Therefore, the proposed method is suitable for severely degraded LFP samples. A feasible option is to use higher concentration solutions, which will need more investigations.

TGAs of s-LFP, LFP-hydro, LFP-hydro-TA and re-LFP were conducted (Fig. S6). Except for re-LFP, the other three samples lost weight at 200 °C, which is attributed to the release of adsorbed water. In the range 200–300 °C, the other three samples kept losing weight due to the oxidation of the carbon or organic composition, but the weight of re-LFP remained unchanged. When the temperature was increased to 300–400 °C, the weight of re-LFP increased, but at a slower rate than the other three samples, due to simultaneous carbon oxidation and oxygen absorption. Equation 6 can describe the whole reaction when the carbon-coated LiFePO_4_ (re-LFP) was heated in air.

6 $${\text{xC }} + {\text{ 12LiFePO}}_{{4}} + \, \left( {{3} + {\mathrm{x}}} \right){\mathrm{O}}_{{2}} \to {\text{ 4Li}}_{{3}} {\mathrm{Fe}}_{{2}} \left( {{\mathrm{PO}}_{{4}} } \right)_{{3}} + {\text{ 2Fe}}_{{2}} {\mathrm{O}}_{{3}} + {\text{ xCO}}_{{2}}$$

The final remaining mass ratio can be calculated to be $\frac{{4M_{{\left( {{\mathrm{Li}}_{{3}} {\mathrm{Fe}}_{{2}} \left( {{\mathrm{PO}}_{{4}} } \right)_{{3}} } \right)}} + 2M_{{\left( {{\mathrm{Fe}}_{{2}} {\mathrm{O}}_{{3}} } \right)}} }}{{xM_{\left( C \right)} + 12M_{{\left( {{\mathrm{LiFePO}}_{{4}} } \right)}} }} = 0.9996$. In this regard, the carbon content can be calculated by solving this equation, where *M*_(A)_ refers to the molar mass of A. The carbon content of re-LFP was calculated to be 5.21%.

As for the other three LFP samples, taking all residual (water, carbon and organic composition) into account as a unity, the whole reaction can be describes by Eq. 7:

7 $${\text{Residual }} + {\text{ 12LiFePO}}_{{4}} + {\text{ 3O}}_{{2}} \to {\text{ 4Li}}_{{3}} {\mathrm{Fe}}_{{2}} \left( {{\mathrm{PO}}_{{4}} } \right)_{{3}} + {\text{ 2Fe}}_{{2}} {\mathrm{O}}_{{3}} + {\text{ Residual }}\left( {\mathrm{g}} \right)$$

The volatile residual ratio of s-LFP is calculated to be 5.64% from equation $\frac{{4M_{{\left( {{\mathrm{Li}}_{{3}} {\mathrm{Fe}}_{{2}} \left( {{\mathrm{PO}}_{{4}} } \right)_{{3}} } \right)}} + 2M_{{\left( {{\mathrm{Fe}}_{{2}} {\mathrm{O}}_{{3}} } \right)}} }}{{12M_{{\left( {{\mathrm{LiFePO}}_{{4}} } \right)}} + m_{{\left( {{\mathrm{residual}}} \right)}} }} = 0.9914$. After TA coating treatment, the volatile residual ratio is increased to 7.85%, which proves the succuss TA coating.

XRD measurements were made at different temperatures on s-LFP and re-LFP to detect phase changes at high temperatures (Fig. 1e, f). Overall, LFP went through an oxidation reaction to produce Li_3_Fe_2_(PO_4_)_3_ and Fe_2_O_3_, which was confirmed by XRD of samples heated at 800 °C (Fig. S7). More detailed XRD patterns of s-LFP and re-LFP are shown in Fig. S8, in which the diffraction peak of (020), (301) and (311) lattice planes of LFP phase are highlighted. When s-LFP was heated to 400 °C, the three peaks shifted to a lower degree and the crystal structure begins to collapse. This phenomenon appeared at 450 °C when heating re-LFP, indicating re-LFP is thermally more stable than s-LFP.

The carbon layers of all samples were characterized by Raman spectroscopy (Fig. 1g). The *D* (1350 cm^−1^) and *G* peaks (1582 cm^−1^) are features of carbon. The former corresponds to the A_1g_ (lattice vibration of amorphous carbon) mode associated with the breaking of the symmetry at the edge of the graphite sheet, while the latter corresponds to the E_2g_ phonon scattering mode of the *sp*^2^ carbon atoms in an ideal graphite single crystal. The intensity ratio of the *D* to *G* peaks (*I*_D_/*I*_G_) is used to evaluate the degree of graphitization [36–38]. In addition, the increase in full width at half height (FWDH) and the redshift of the *G* peak in s-LFP indicate that a large number of defects were generated in the carbon layer during previous use [37]. The *I*_D_/*I*_G_ of re-LFP is much lower than that of s-LFP, even lower than c-LFP, suggesting a higher degree of graphitization of the restored carbon coating layer, which increases the electroconductivity.

**Fig. 2:**
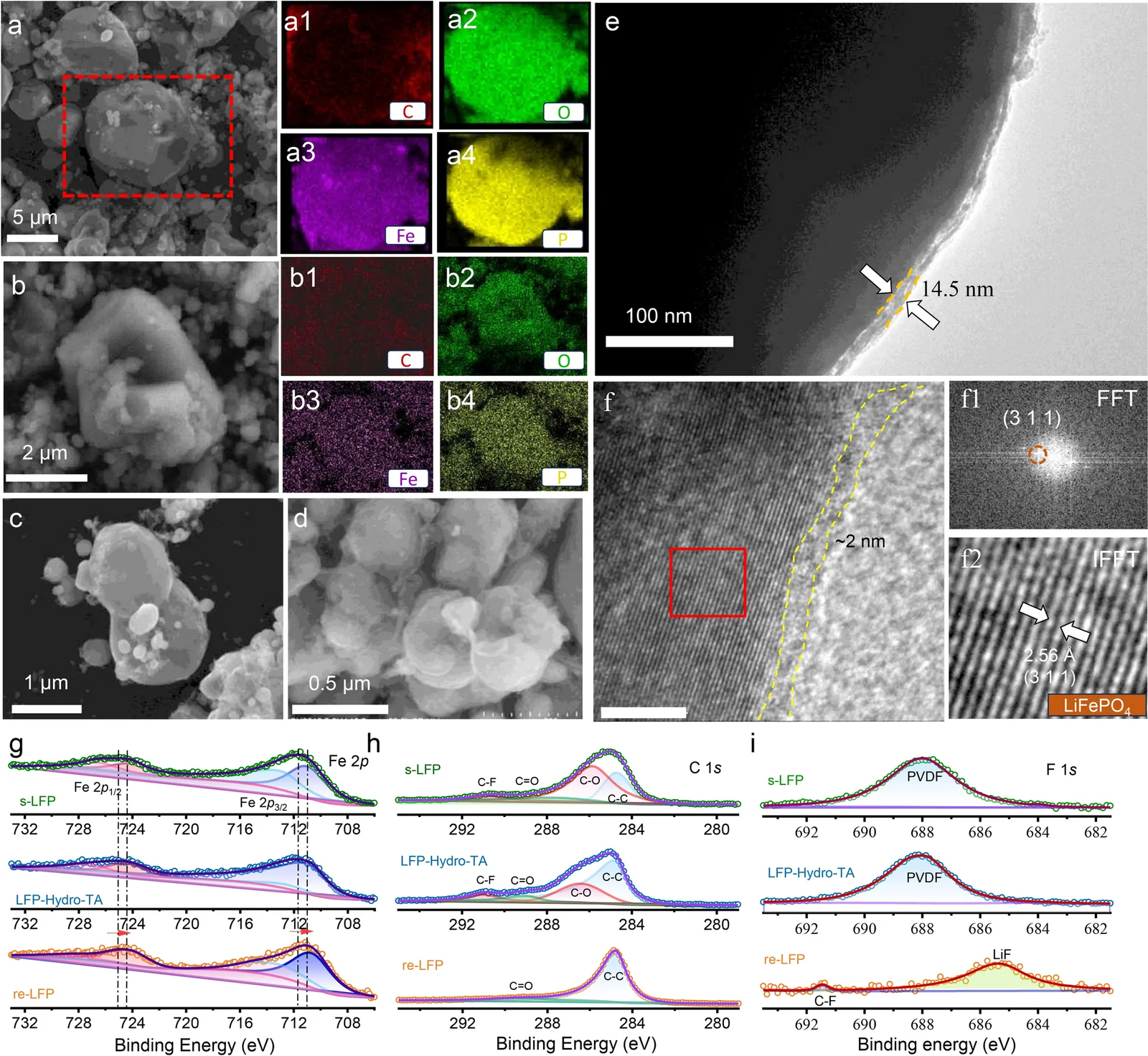
Microscopic structure of different LFP samples. SEM images of **a** s-LFP and **b** re-LFP and their corresponding elemental maps. SEM images of **c** s-LFP and **d** LFP-Hydro-TA. HRTEM images of **e** LFP-Hydro-TA and **f** re-LFP. Figures 1f and 2f are respectively the FFT and IFFT for the selected area in Fig. 2f. **g** Fe 2*p*, **h** C 1*s* and **i** F 1*s* XPS spectra of s-LFP, LFP-Hydro-TA and re-LFP

The microscopic structure of different LFP samples was observed by SEM and TEM. SEM images of s-LFP and re-LFP particles are shown in Fig. 2a, b. Elemental mapping was obtained by energy-dispersive X-ray spectroscopy and shows that P, Fe and O are evenly distributed within the particles of both s-LFP (Figs. 2a and 4a) and re-LFP (Figs. 2b and 4b). However, the elemental map for carbon (Fig. a1) of s-LFP showed that the coating layer is uneven. The O/Fe atomic ratio of LFP-Hydro-TA was about 6.9, obviously higher than that of s-LFP (O/Fe = 4.45) and re-LFP (O/Fe = 2.48) (Fig. S9), suggesting the presence of a cathode/electrolyte interface (CEI) or residual H_2_O on the s-LFP and the successful TA coating in LFP-Hydro-TA, respectively. In addition, a damaged layer of conductive carbon is seen to be attached to the surface of s-LFP particles, as shown in Fig. 2c, which may cause a higher impedance. Damaged crystal structures of the FePO_4_ and Fe_2_O_3_ phases are mostly detected on the surface of s-LFP particles (Fig. S10). Therefore, repairing the s-LFP involves restoring the carbon coating layer and repairing the crystal damage.

After stirring LFP-hydro for a short time in the TA solution, it is found that a layer of TA about 14.5 nm thick is evenly and tightly coated to the surface of the particles (Fig. 2e). This observation was further corroborated by a HRTEM image of LFP-hydro-TA (Fig. S11). After hydrothermal relithiation, TA coating and heat treatment, the re-LFP had a crystalline LFP phase and uniform carbon coating layer (Fig. 2f), which confirms the effectiveness of our repair method. The fast Fourier transform (FFT) and inverse fast Fourier transform (IFFT) images show the (311) lattice plane of LFP, indicating its good crystallinity.

To investigate the differences in the surfaces of the different LFP samples, XPS analyses were performed. As shown in Fig. 2g, the Fe 2*p* spectra shows a higher valence state in s-LFP, which is consistent with the TEM results. After hydrothermal treatment and TA coating, the Fe 2*p* peak shifted to a lower binding energy, proving that the Fe(III) is reduced to Fe(II), corresponding to the results of TEM and XRD. It is obvious from Fig. 2h that the intensity of the C–O peak in the C 1*s* spectra increases, illustrating that the carbon coating layer of s-LFP is oxidized due to electrochemical use or the pretreatment process. After hydrothermal treating and TA coating, a C = O peak appears due to the large amount of C = O in the TA molecules, proving the coating of the LFP particles with TA. This is consistent to the TEM result, as well as the O 1*s* spectra in Fig. S12a. In addition, residual PVDF binder attached to the surface of the s-LFP particles is identified, as shown by the peak at around 688 eV in the C 1*s* spectra in Fig. 2h and F 1*s* spectra in Fig. 2i. After the heat treatment, the peak at around 688 eV disappears and a peak at around 685 eV appears, indicating the decomposition of PVDF and formation of LiF on the surface of particles. This result agrees with the full XPS spectra of the three LFP samples shown in Fig. S13. The F 1*s* peak of re-LFP was almost invisible compared to that of the other two LFP samples in the full XPS spectra (Fig. S13), implying that most of the F was released during the heat treatment. The P 2*p* spectra (Fig. S12b) show a typical P–O peak at around 134 eV, indicating that the PO_4_ framework remained intact during the entire repair process.

### Electrochemical Performance and Kinetics of LFP

**Fig. 3:**
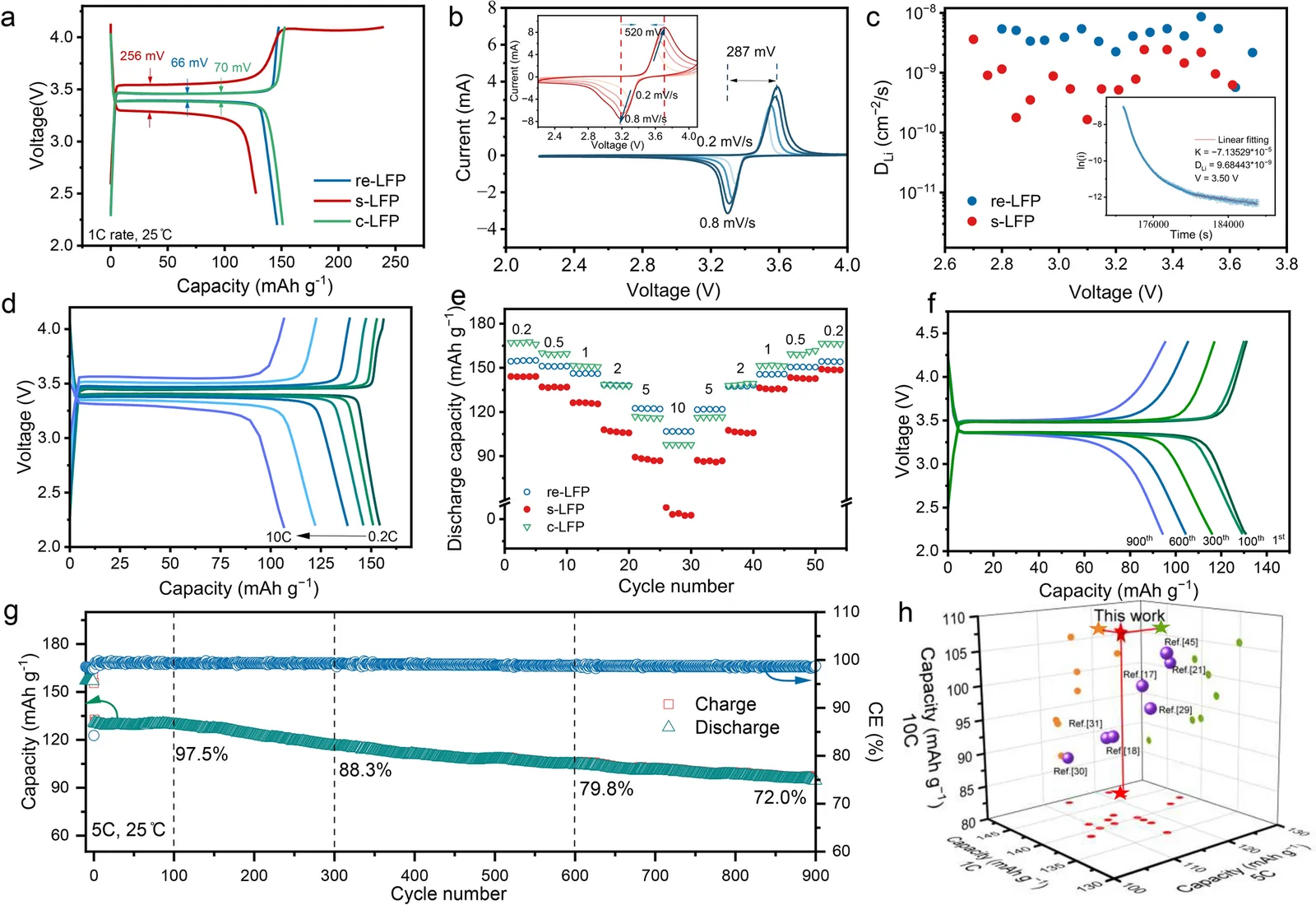
Electrochemical characterization of different LFPs at room temperature. **a** GCD profiles of s-LFP, re-LFP and c-LFP. **b** CV curves of re-LFP and s-LFP (inset). **c** Apparent *D*_Li_ of re-LFP and s-LFP calculated from the PITT results and the ln**i**-t curve of re-LFP at 3.5 V from the PITT tests (inset). **d** GCD profiles of re-LFP at different rates. **e** Rate performance of s-LFP, re-LFP and c-LFP. **f** GCD curves at different cycles at 5C and **g** the corresponding cycling performance of re-LFP. **h** Reversible capacity comparison with other published studies [17, 18, 21, 29–31, 45]

To evaluate the effect of the heat treatment and TA coating, the s-LFP, LFP-Hydro, LFP-Hydro-HT and re-LFP were electrochemically tested. GCD curves of half cells are shown in Figs. 3a and S14. The GCD profile at 1C (1C = 170 mA g^−1^) of s-LFP shows a high voltage gap of over 256 mV and an extremely low Coulombic efficiency (CE) due to the unstable CEI [39–42]. The other three samples all reached ideal reversible capacities exceeding 140 mAh g^−1^, among which re-LFP had the highest reversible capacity of 146 mAh g^−1^ at the 1C rate. In comparison, LFP-Hydro shows a high voltage gap of over 358 mV at 1C. The initial discharge capacity of LFP-Hydro was only 120 mAh g^−1^ at 2C rate, and the voltage gap became wider during the cycling (Fig. S15a, b). With a post heat treatment, LFP-Hydro-HT displays a decreased voltage gap of 150 mV at 1C (Fig. S14), with a relatively good rate performance (Fig. S15c). A comparison of the cycling performance of LFP-Hydro and LFP-Hydro-HT is shown in Fig. S16 which shows that they had respective capacity retentions of 92.6% and 83.8% after 500 cycles at 0.5C. In short, heat treatment improves the electrochemical performance to some extent; however, the CEs of both were below 99% at all rates, indicating a severe side reaction on the surface.

In this context, we introduced a TA coating process to reconstruct the surface of LFP particles. The charge–discharge voltage gap of re-LFP further decreases to 66 mV at 1C, as shown in Fig. 3a, showing that the carbon coating layer lowers the impedance of these materials, and it displays a capacity retention of 98.7% at 100 cycles. The re-LFP delivers an initial capacity of 138 mAh g^−1^ at the 2C rate, with a capacity retention of 92.1% at 200 cycles (Fig. S17). In comparison, the s-LFP becomes unstable at around 120^th^ cycles (Fig. S18).

CV curves at different voltage sweep rates ranging from 0.2 to 0.8 mV s^−1^ are shown in Fig. 3b. The voltage gap between the oxidation and reduction peaks at 0.8 mV s^−1^ is only 287 mV, which is much smaller than that of s-LFP (520 mV, Fig. 3b insert). To discover the origin of the improved electrochemical performance, the PITT was used to evaluate *D*_Li_ considering the two-phase reaction for LFP [43, 44], as described in Eq. 8:

8 $$\ln \left( i \right) = \ln \left( {2\Delta QD_{{{\mathrm{Li}}}} /L^{2} } \right) - \left[ {\pi^{2} D_{{\mathrm{Li/}}} \left( {4L^{2} } \right)} \right]$$

where *i* refers to the equilibrium electrode current, Δ*Q* is the electric charge during (de)lithiation, and *L* is the depth of the active materials on the collector. Hence, the value of apparent *D*_Li_ can be calculated from the linear slope of a plot of ln(*i*)-*t* versus voltage, as shown in the insert in Fig. 3c. The detailed PITT testing condition and calculation is given in the Supporting Information. The apparent *D*_Li_ for re-LFP delithiation calculated from the PITT results is of the order of 10^−9^, which is about one-order higher than that for the s-LFP cathode (10^−10^) and is the highest reported to the best of our knowledge [23], which explains the improved performance.

Thanks to this improvement, re-LFP has an excellent rate performance, as shown in Fig. 3d, e. It has reversible capacities of 122.5 and 106.7 mAh g^−1^ at 5C and 10C, respectively, which are even higher than that of c-LFP (116.4 and 97.7 mAh g^−1^ at 5C and 10C, respectively). Its long-term cycling performance at 5C is shown in Fig. 3f, g, which shows that the capacity retention at 5C was 97.5%, 88.3%, 82.3%, 79.8% and 72.0% after 100, 300, 500, 600 and 900 cycles, respectively. In addition, the voltage gap of the GCD profiles at different cycles does not show a significant difference, implying a stable reaction interface during long-term cycling. The final reversible capacity is about 94.4 mAh g^−1^ after 900 cycles, with a CE above 99.5%. Besides, re-LFP has a near 70% capacity retention after 900 cycles at 4C, 6C, 8C and 10C (Fig. S19), much better than s-LFP (< 38% capacity retention after 500 cycles at 4C, Fig. S20). Such a high-rate performance also surpasses that of other regenerated LFPs repaired by direct methods (Fig. 3h–j and Table S6).

**Fig. 4:**
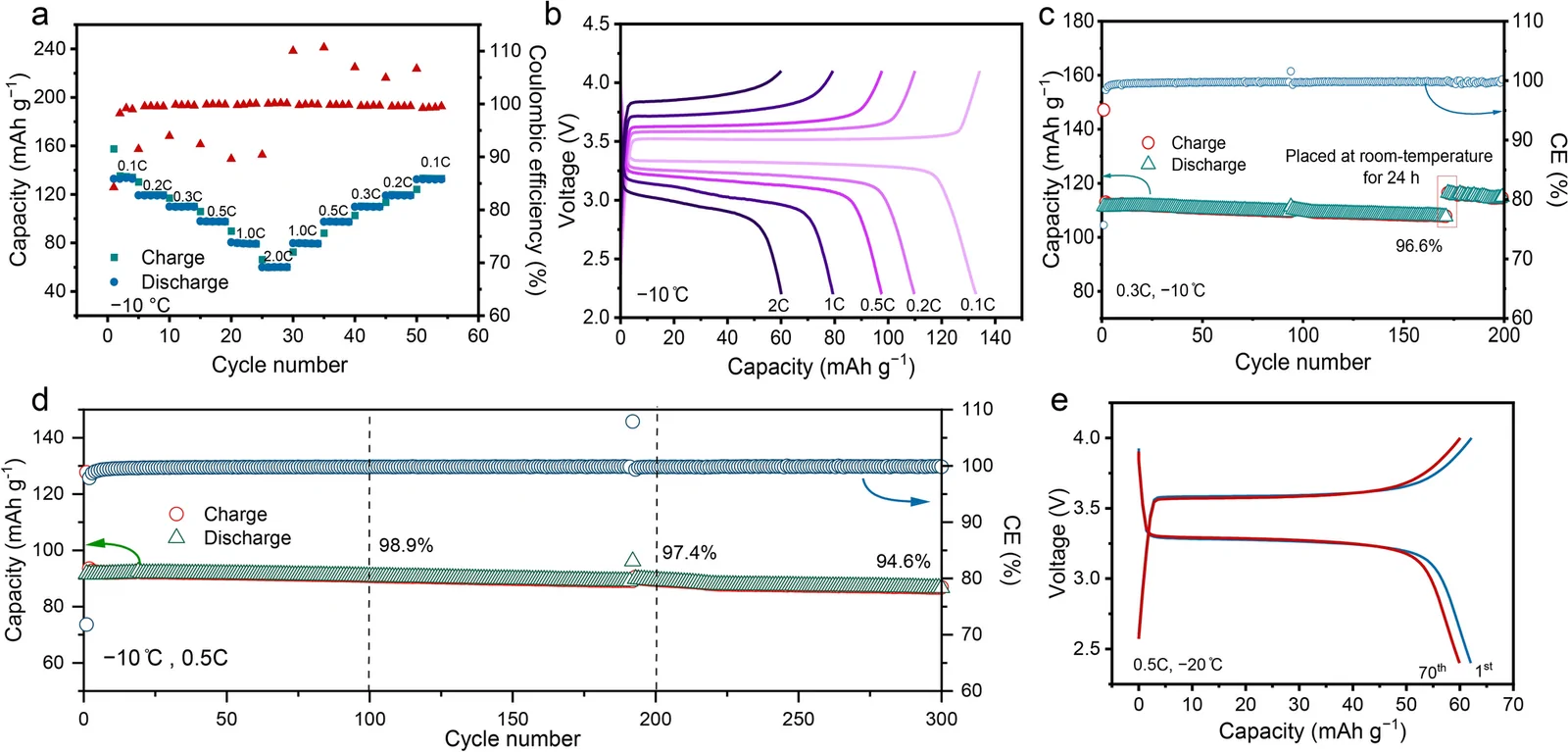
Electrochemical characterization at low temperatures. **a** Rate performance of re-LFP, **b** its corresponding GCD profiles at different rates and its cycling performance at − 10 °C at **c** 0.3C and **d** 0.5C. **e** Stabilized GCD profile of re-LFP at 0.5C and − 20 °C

The low-temperature electrochemical performance was also examined on the re-LFP. At a temperature of − 10 °C, it has reversible capacities of 132.8, 119.1, 109.8, 97.7, 79.5 and 60.1 mAh g^−1^ at 0.1C, 0.2C, 0.3C, 0.5C, 1C and 2C, respectively (Fig. 4a, b). The capacity at the lower rates could be fully recovered by higher-rate charging–discharging and the CEs are close to 100% across all rates. It is worth noting that the capacity retention reached 96.6% at − 10 °C after 175 cycles (0.3C), and the reversible capacity increased rapidly after returning it to room temperature for one day (Fig. 4c). As for the cycling performance, re-LFP, at − 10 °C and 0.5C, had capacity retentions of 98.9%, 97.4% and 94.6% after 100, 200 and 300 cycles, respectively, with CEs approaching 99.9%, as shown in Fig. 4d. At − 20 °C, the reversible capacity at 0.5C reached 61 mAh g^−1^, and there is a small amount of capacity loss after the first 70 cycles with a CE close to 100% (Figs. 4e and S21). The exceptional low-temperature electrochemical performance reported here surpasses most reported values, highlighting the suitability of re-LFP for low-temperature applications (Table S6).

### Characterization of Repaired LFP After Cycling

**Fig. 5:**
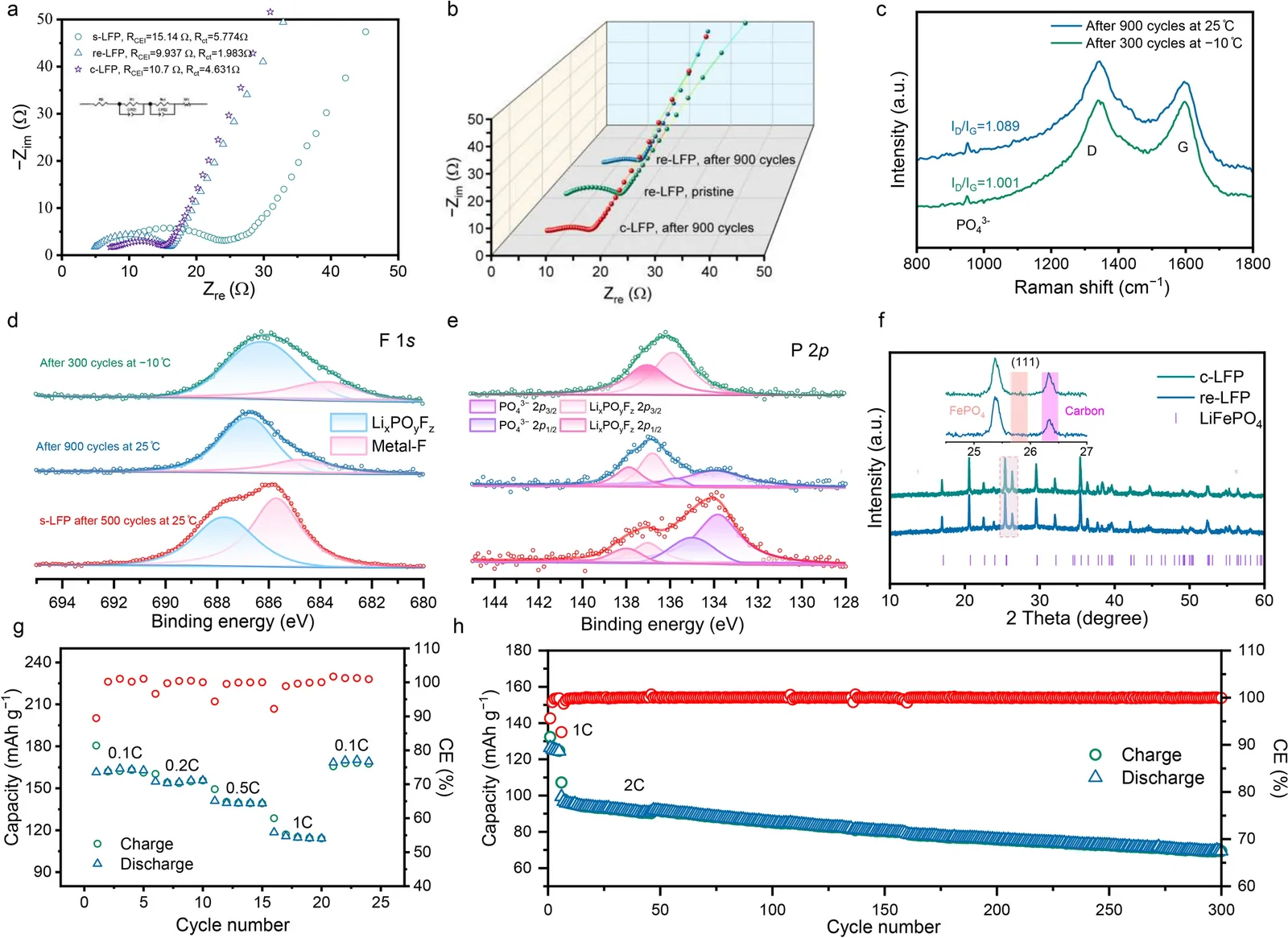
Characterization of different LFP samples. **a** Nyquist plots of half cells assembled with s-LFP, re-LFP and c-LFP materials after electrochemical activation. **b** Nyquist plots of half cells assembled with re-LFP and c-LFP after 900 cycles at 5C. **c** Raman spectra of re-LFP after cycling at 25 °C and − 10 °C. **d** F 1*s* and **e** P 2*p* XPS spectra of s-LFP and re-LFP electrodes cycled at 25 °C and re-LFP cycled at − 10 °C. **f** XRD patterns of recollected re-LFP and c-LFP after 900 cycles. **g** The rate performance and **h** long-term cycling performance of re-LFP assembled with solid-state electrolyte and lithium metal anode

After the electrochemical evaluation, we examined the changes in the recycled LFP by Raman spectroscopy, XPS and SEM. Figure 5a shows the Nyquist plots and the equivalent circuit fitting results for s-LFP, re-LFP and c-LFP electrodes after electrochemical activation. The resistance of the CEI (*R*_CEI_) of re-LFP is significantly reduced to 9.94 Ω, which is comparable to that of c-LFP (*R*_CEI_ = 10.7 Ω), and its charge transfer resistance (*R*_ct_) is also significantly reduced to 1.98 Ω, outperforming that of c-LFP (*R*_ct_ = 4.63 Ω). After 10 cycles of electrochemical activation, these *R*_CEI_ and *R*_ct_ values decreased to 5.17 and 1.55 Ω, respectively (Fig. S22), suggesting improved charge transfer kinetics in first few cycles. After 500 and 900 electrochemical cycles at 5C, the respective values of *R*_ct_ increased slightly to 13.89 and 14.96 Ω. This increased resistance is comparable to that of c-LFP, indicating that re-LFP retains high-rate electrochemical activity during prolonged cycling (Fig. 5b). After 300 cycles at 0.5C and − 10 °C, *R*_CEI_ and *R*_ct_ increased to 39.47 Ω and 52.7 Ω, respectively (Fig. S23), in agreement with the slightly worse performance at low temperature.

Cycled cells were dissembled to evaluate the surface chemical components by Raman spectroscopy and XPS. Compared to the pristine sample, the *I*_D_/*I*_G_ of re-LFP (0.890 in Fig. 1g) cycled at room and low temperatures (− 10 °C, Fig. 5c) increased to 1.089 and 1.001, indicating a lower degree of graphitization of carbon coating layer caused by electrochemical cycling. In XPS analysis [39], the F 1*s* spectra was around 687 and 685 eV (Fig. 5d), which can be assigned to Li_x_PO_y_F_z_ and metal-F, respectively. This result is consistent with P 2*p* spectra in Fig. 5e, where the 2*p*_3/2_-2*p*_1/2_ doublet at 136.5 − 138 eV is assigned to Li_x_PO_y_F_z_. On the surface of re-LFP cycled at 25 and − 10 °C, less metal fluoride (LiF or FeF_x_) is produced compared with the cycled s-LFP. In addition, a more pronounced P 2*p*_3/2_-2*p*_1/2_ doublet at 133.4 − 135 eV can be detected in the cycled s-LFP electrode, which is assigned to PO_4_^3−^. This peak is significantly weaker in the cycled re-LFP at both 25 and − 10 °C, indicating the suppressed decomposition of LiPF_6_ during electrochemical cycling [23, 46]. Our findings indicate the successful fabrication of a stable interface on the regenerated materials, resulting in better high-rate and low-temperature capabilities.

Figure 5f shows the XRD patterns of recollected re-LFP and c-LFP after 900 cycles, compared with that of the standard LFP. From the inset, diffraction peaks at around 25.8°, corresponding to FePO_4_ (111) are detected for the c-LFP but almost invisible for the re-LFP. The XRD refinement of both cycled electrodes are presented in Fig. S24. The results show that the phase ratio of FePO_4_/LFP of cycled c-LFP is 0.095, which is higher than that of cycled re-LFP electrode (0.048). These results suggest the re-LFP has a lower Li loss during cycling.

Considering the fast ion migration kinetics and stable CEI of the re-LFP, we have explored its potential use in solid-state Li metal batteries [47]. As shown in Figs. 5g, h and S25, the discharge capacities are 162.2, 155.6, 139.3 and 114.7 mAh g^−1^ at 0.1C, 0.2C, 0.5C and 1C, respectively, and the discharge capacity at 0.1C is fully recovered after cycling at higher rates, demonstrating excellent electrochemical reversibility of re-LFP in a solid-state electrolyte battery. The battery also has a capacity retention over 70% after 300 cycles at 2C, highlighting its excellent cycling stability. Therefore, the use of re-LFP in solid-state batteries is also feasible. Further investigation of the interface between the different LFPs and solid-state electrolytes is ongoing.

### Environmental–Economic Analysis

To evaluate the practicability of our direct regeneration method, the environmental and economic effect of recycled s-LFP was analyzed using the EverBatt model (2023 version, Argonne National Laboratory), in comparison with the hydrometallurgical and other direct regeneration methods.

**Fig. 6:**
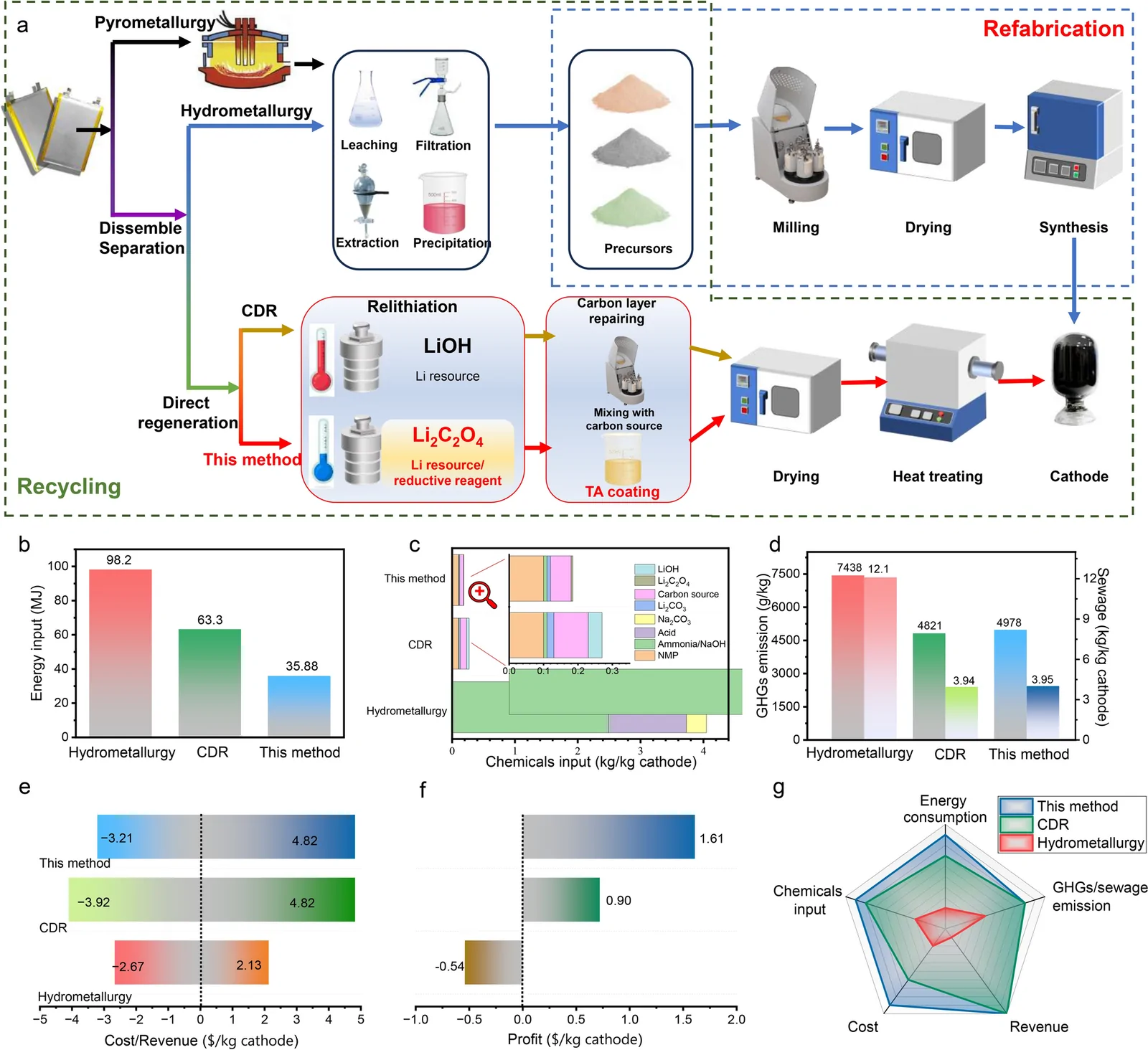
Environmental and economic analysis for hydrometallurgical, conventional direct recycling methods and this method. **a** Closed-loop flowchart of recycling and cathode refabrication. **b** Energy consumption, **c** chemicals input and **d** GHGs emissions per kg of s-LFP. **e** Cost/revenue and **f** profit per kg of s-LFP. **g** Comprehensive comparison of the three methods, with the degree of superiority represented by the line segments of the hexagon from the center to each of the six vertices

Figure 6a illustrates the recycling flowchart of spent LIBs by pyrometallurgical, hydrometallurgical and direct regeneration methods. The pyrometallurgical method offers the advantage of a simple process without LIB disassembly and component separation. Despite its convenience, it is not suitable for recycling spent LFP-type LIBs due to its low valuable products [48]. The hydrometallurgical method involves dissembling and separating process, after which the spent LIB cathode materials undergo leaching, filtration, extraction and precipitation process to obtain valuable metal salts [49–51].

Typically, the recycled alloys obtained by pyrometallurgy are further processed by hydrometallurgical methods to leach, extract, separate and precipitate valuable metals. For a closed-loop recycling process, the metal salts such as Li_2_CO_3_, transition metal sulfates worked as precursors for the refabrication process to produce brand new cathode materials. Concretely, for recycling LFP, most of the hydrometallurgical methods are focused on selective recovering lithium element from spent LFP, such as acid leaching H_2_SO_4_, citric acid [52–54] and oxidant leaching (Na_2_S_2_O_8_, (NH_4_)_2_S_2_O_8_) [55, 56]. New LFP can be refabricated from the remaining FePO_4_ and recovered lithium salts, as shown in Fig. 6a. In contrast, direct regeneration recycling avoids destruction of the crystalline phase and element recombination, providing a more effective way to obtain cathode materials with a nearly perfect crystalline structure.

Assuming a feedstock of 50,000 tons s-LFP per year, three recycling models were established. Detailed descriptions of the simulation procedures were provided in the experimental section, with reference to published works as the basis for the modeling [52, 57]. For the method reported here, the relithiation temperature was lower because of the thermodynamic favorable relithiation reaction, and the energy consumption for recycling 1 kg of s-LFP black mass was reduced from 98.2 to 35.9 MJ (Fig. 6b). Regarding the chemicals needed for the three different recycling procedures (Fig. 6c), hydrometallurgical recycling consumed substantially larger quantities during the leaching, extraction and precipitation processes. It makes sense that nearly 95% of the energy used for hydrometallurgical recycling comes from the upstream production of input chemicals. Greenhouse gases (GHG) emission caused by hydrometallurgical recycling was approximately 7,438 g kg^−1^ of LFP black mass, and was reduced to about 4,900 g kg^−1^ for the direct regeneration method. In addition, the waste produced during recycling process by hydrometallurgical method was nearly three times than that caused by direct regeneration. Hydrothermal relithiation was chosen as our conventional direct recycling (CDR) procedure. The waste produced by our method and by CDR was nearly identical. The GHG emission in our method was slightly higher than in the CDR method, potentially because of the carbon dioxide produced during relithiation, as described in Eqs. 1–4. The economic analysis is illustrated in Fig. 6e, f. While the hydrometallurgical recycling of s-LFP incurred the lowest costs, the revenue generated from the resulting products was insufficient to cover the expense. Moreover, the volatile price of lithium salts poses tremendous challenges for hydrometallurgical recycling industries. Although recycling s-LFP by direct regeneration methods involves a relatively high cost, it remained profitable due to the valuable LFP output. Compared to CDR methods, our method has a cost advantage, with the profit generated by recycling 1 kg of s-LFP black mass being approximately $1.61. Therefore, the proposed direct regeneration method for s-LFP cathode materials is economically viable and environmental friendly (Fig. 6g).

## Conclusions

The s-LFP material was regenerated by an integrated repair of the olivine crystal structure and coating surface, combining low-temperature hydrothermal relithiation, TA coating and short-time annealing. Comprehensive material characterization and electrochemical measurements demonstrated that the re-LFP facilitates Li-ion diffusion and improves structural reversibility, with a stable interface with the electrolyte that prevents side reactions and decreases interface impedance. The superfast Li migration dynamics give an excellent high rate (5C and 10C) and low-temperature (− 10 and − 20 °C) performance and excellent compatibility with a solid-state electrolyte. Based on the Everbatt model, the proposed method demonstrated great economic and environmental benefits over the traditional hydrometallurgical method and other direct regeneration techniques. This work offers a promising way to develop high performance LFP from spent materials through an economic and environmentally-friendly approach.

## Supplementary Information

Below is the link to the electronic supplementary material.

Supplementary file1 (DOCX 32020 KB)
