# Supplementary material for: Direct Repair of the Crystal Structure and Coating Surface of Spent LiFePO4 Materials Enables Superfast Li-Ion Migration
Source: Nanomicro Lett. 2026 Jan 5;18:137. doi: 10.1007/s40820-025-01980-1 (PMC12765765; doi:10.1007/s40820-025-01980-1)
Supplement: Supplementary file 1 — Supplementary file1 (DOCX 32020 KB) [file 40820_2025_1980_MOESM1_ESM.docx]

Supporting Information for

**Direct Repair of the Crystal Structure and Coating Surface of Spent LiFePO_4_ Materials Enables Superfast Li-Ion Migration**

Yuanqi Lan^1, 2^*^#^*, Jianfeng Wen^1^*^#^*, Yatian Zhang^1^, Xuexia Lan^3^, Tianyi Song^1^, Jie Zhu^1^, Jing Peng^3^, Wenjiao Yao^1, 2,3^ *, Yongbing Tang^1, 2,^ *, Hui-Ming Cheng^2, 3^

^1^ Advanced Energy Storage Technology Research Center, Shenzhen Institutes of Advanced Technology, Chinese Academy of Sciences, Shenzhen 518055, P. R. China

^2^ University of Chinese Academy of Sciences, Beijing 100049, P. R. China

^3^ Shenzhen Key Laboratory of Energy Materials for Carbon Neutrality, Shenzhen 518055, P. R. China

^#^Yuanqi Lan and Jianfeng Wen contributed equally to this work.

*Corresponding authors. E-mail: [wj.yao@siat.ac.cn](mailto:wj.yao@siat.ac.cn) (Wenjiao Yao); [tangyb@siat.ac.cn](mailto:tangyb@siat.ac.cn) (Yongbing Tang)

**Supplementary Figures and Tables**





**Fig. S1** Optical images of (**a**) s-LFP and (**b**) re-LFP





Fig. S2 The XRD refinement pattern of the s-LFP black mass


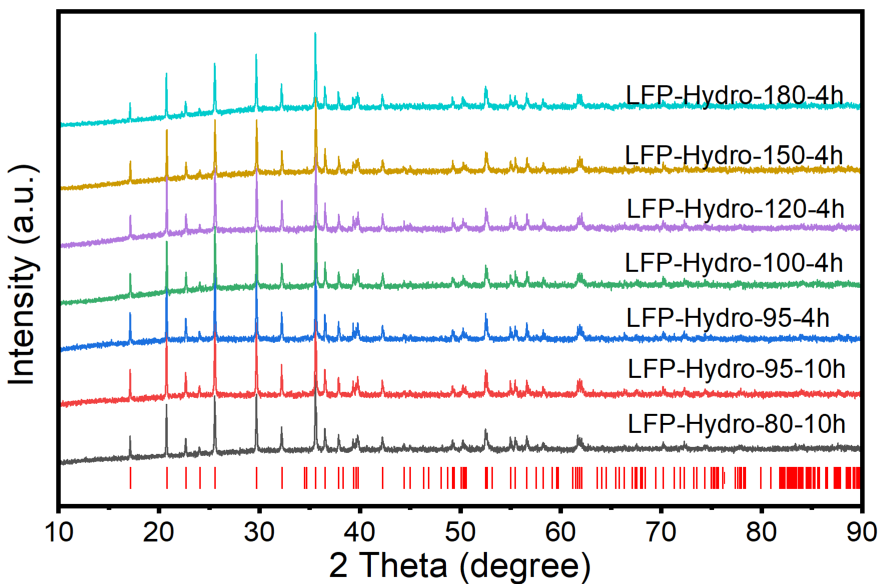


**Fig. S3** The XRD patterns of relithiated LFP sample at different hydrothermal temperature for different time





**Fig. S4** FTIR patterns of re-LFP, LFP-Hydro, LFP-Hydro-TA and s-LFP


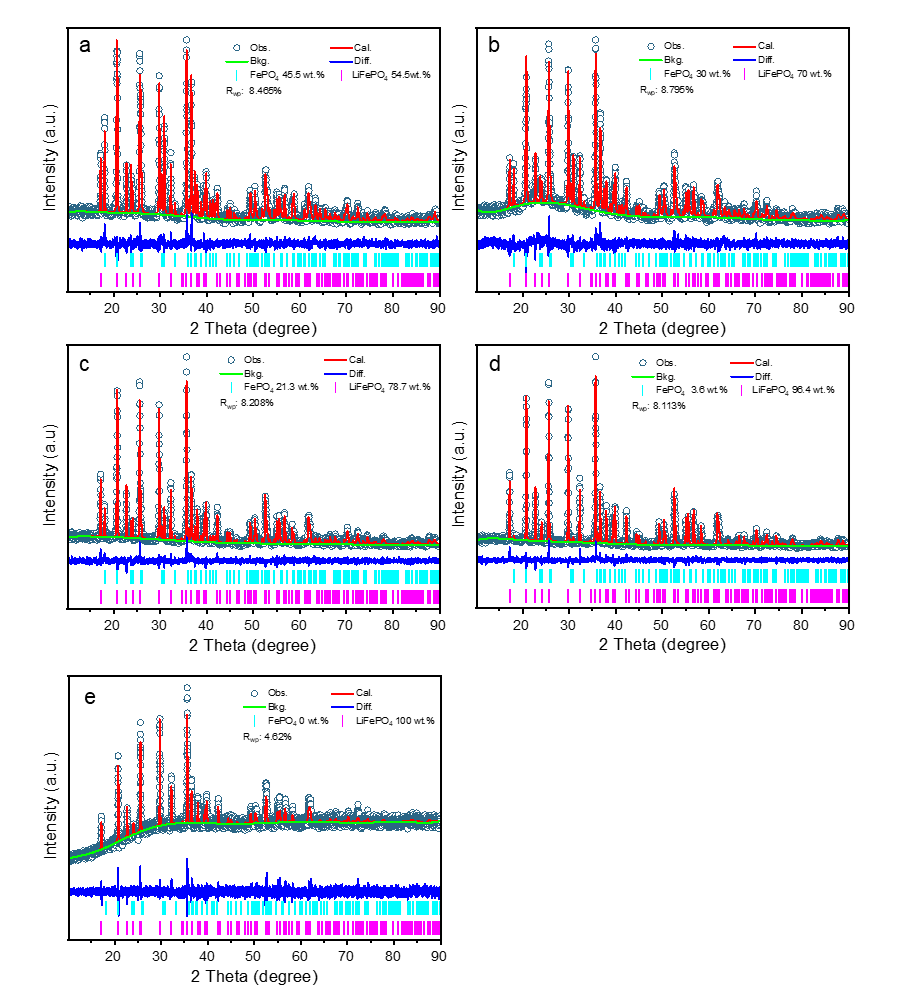


**Fig. S5** XRD refinement results of (**a**) delithiated LFP, and relithiated LFP with hydrothermal reactions for (**b**) once, (**c**) twice (**d**) three times and (**e**) four times





**Fig. S6** TGA of s-LFP, LFP-hydro, LFP-hydro-TA, and re-LFP





**Fig. S7** XRD of LFP residual heated at 800 ℃





**Fig. S8** Detailed XRD patterns of s-LFP (**a**) and re-LFP (**b**) extracted from Fig. 1e, f


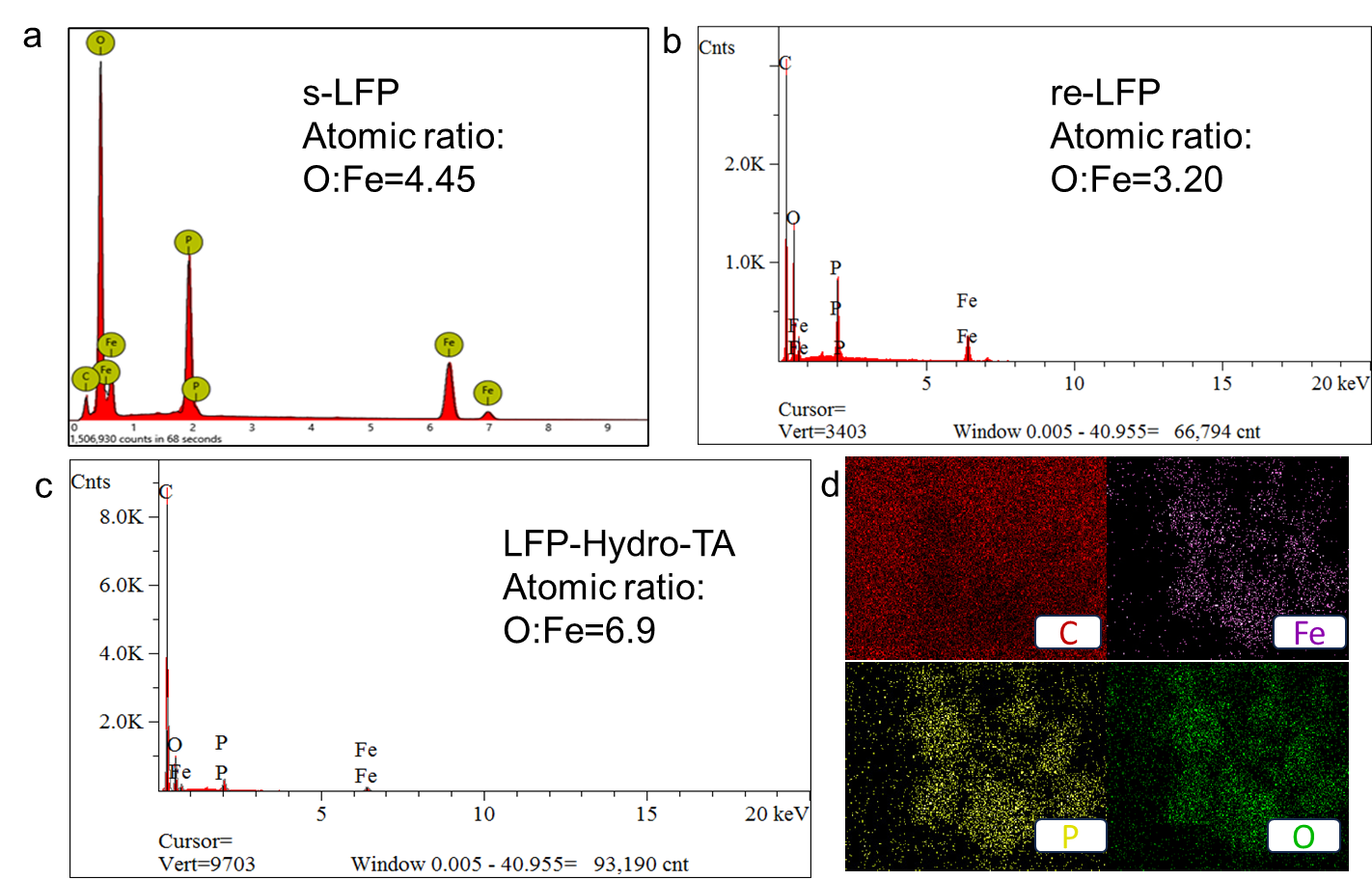


**Fig. S9** EDX analyses of (**a**) s-LFP, (**b**) re-LFP, and (**c**) LFP-Hydro-TA. (**d**) EDX element map of LFP-Hydro-TA





**Fig. S10** TEM image of s-LFP, showing the FePO_4_ and Fe_2_O_3_ phases on the surface





**Fig. S11** TEM image of LFP-Hydro-TA





**Fig. S12** O 1*s* (**a**) and P 2*p* (**b**) XPS spectra of the different LFP samples





**Fig. S13** Full XPS spectra of s-LFP, LFP-Hydro-TA and re-LFP


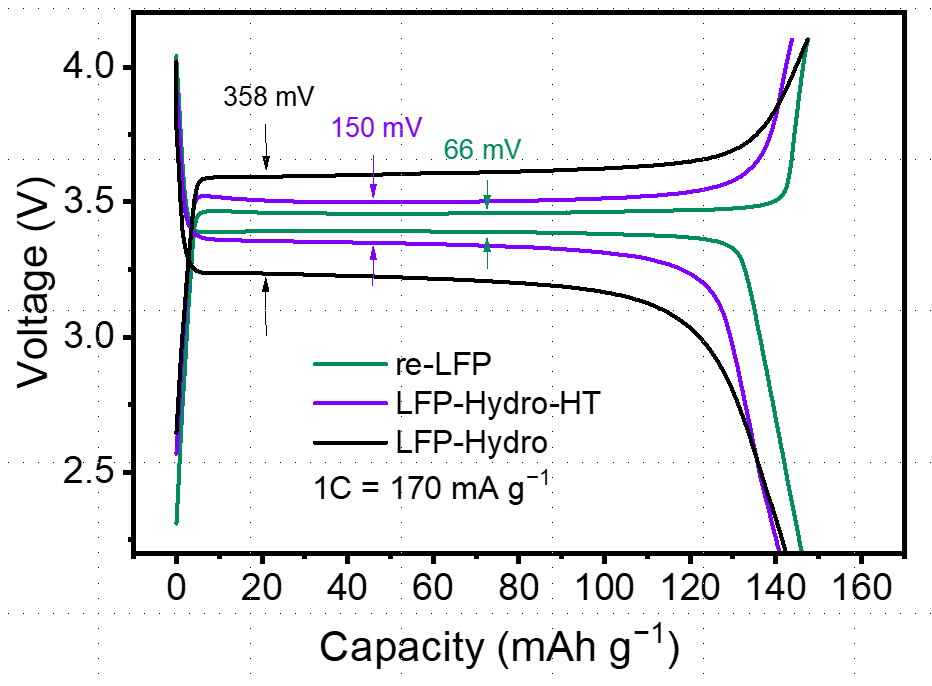


**Fig. S14** Charge-discharge profiles of re-LFP, LFP-Hydro-HT and LFP-Hydro





**Fig. S15** (**a**) Cycling performance of LFP-hydro at 2C rate and (**b**) its charge-discharge profiles at different cycles. (**c**) GCD profile of LFP-Hydro-HT at 0.1C, 0.5C, 1C and 2C, with discharge capacities of 165, 155, 150, 132 mAh g^−1^, respectively





**Fig. S16** Cycling performance of LFP-Hydro-HT and LFP-Hydro at 0.5C


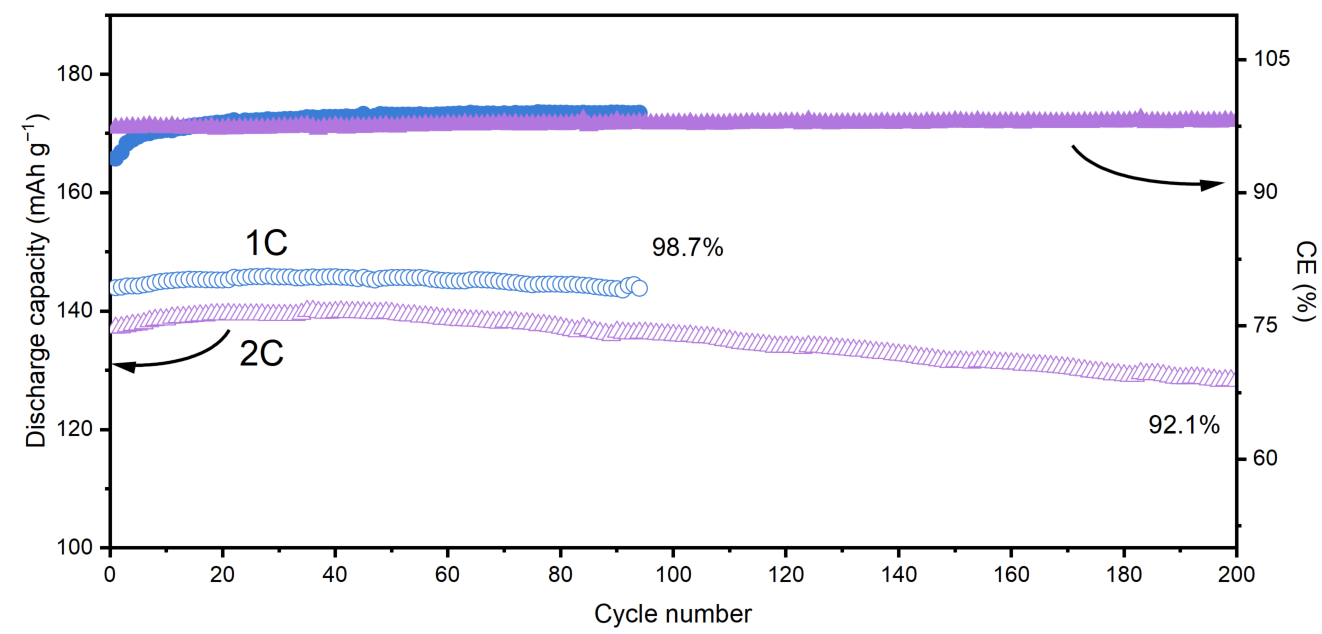


**Fig. S17** Cycling performance of re-LFP at 1C and 2C


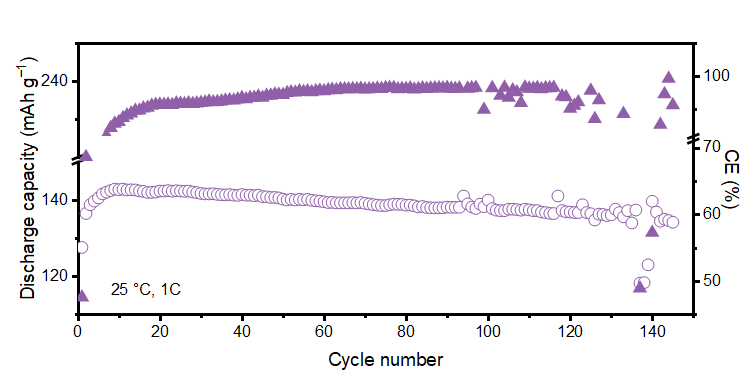


**Fig. S18** Cycling performance of s-LFP at 1C





**Fig. S19** Cycling performance of re-LFP at 4C, 6C, 8C and 10C


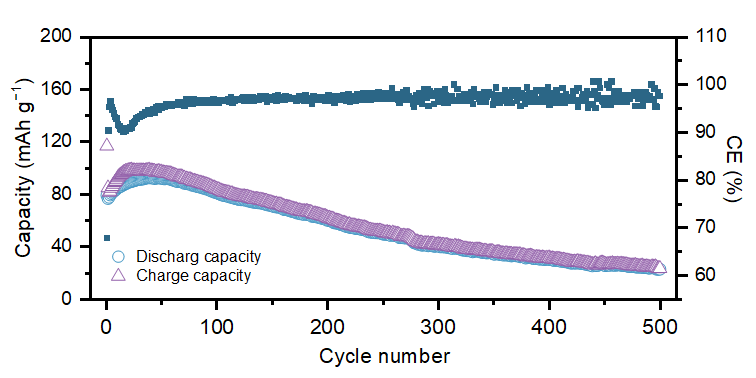


**Fig. S20** Cycling performance of s-LFP at 4C





**Fig. S21** Cycling performance of re-LFP at 0.5C rate, −20 ℃


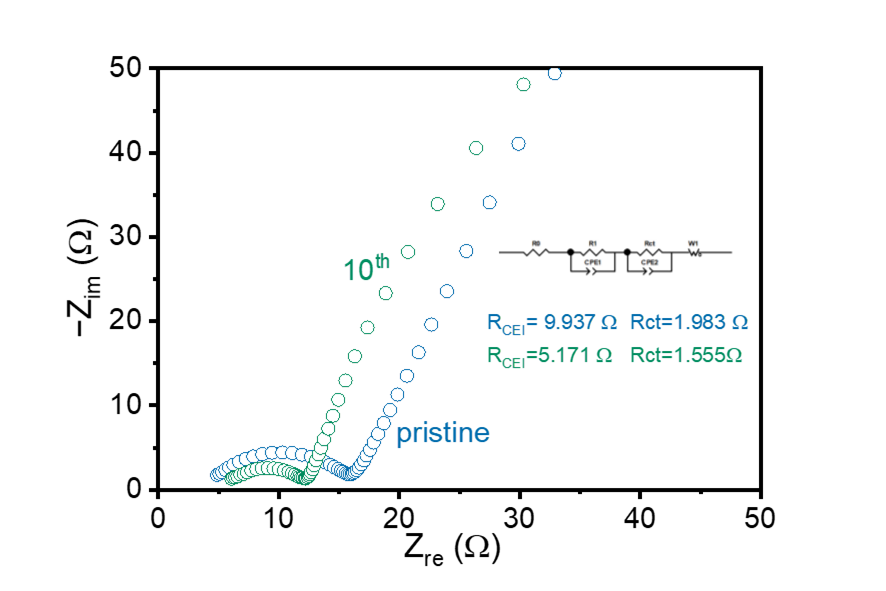


**Fig. S22** Nyquist plots of pristine re-LFP


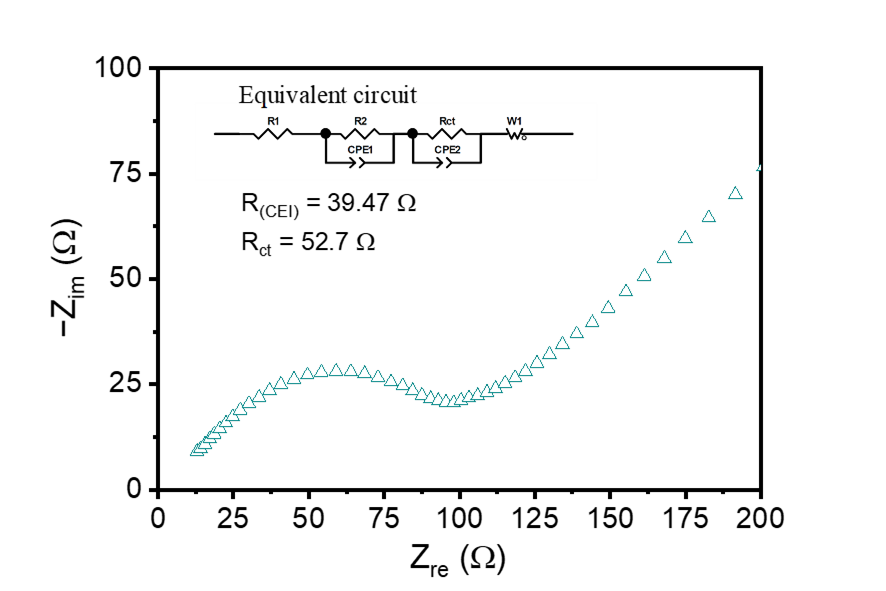


**Fig. S23** Nyquist plot of the cycled re-LFP at −10 ℃ and its equivalent circuit inserted in the figure





**Fig. S24** XRD refinements of **a** cycled c-LFP and **b** cycled re-LFP electrodes





**Fig. S25** Charge-discharge profile of re-LFP in a solid-state electrolyte battery

**Table S1** The chemical input and cost for recycling 1 kg s-LFP black mass used in EverBatt modelling

|  | Input materials | Usage  (kg) | Price  ($) | Products | Price  ($) | Production  (kg) |
| --- | --- | --- | --- | --- | --- | --- |
| Hydrometallurgy | H_2_SO_4_ | 1.24 | 0.01 | FePO_4_  Li_2_CO_3_ | 0.15  10.29 | 0.91  0.266 |
|  | NaOH | 0.01 | 0.35 |  |  |  |
|  | Ammonia | 2.48 | 0.27 |  |  |  |
|  | Na_2_CO_3_ | 0.32 | 0.19 |  |  |  |
| Conventional direct regeneration | LiOH | 0.04 | 15.97 | LiFePO_4_ | 5.07 | 1 |
|  | NMP | 0.1 | 1.32 |  |  |  |
|  | NaOH | 0.01 | 0.35 |  |  |  |
|  | Li_2_CO_3_ | 0.02 | 10.29 |  |  |  |
|  | Sucrose | 0.1 | 1.67 |  |  |  |
| This method | NMP | 0.1 | 1.32 | LiFePO_4_ | 5.07 | 1 |
|  | NaOH | 0.01 | 0.35 |  |  |  |
|  | Li_2_C_2_O_4_ | 0.01 | 111 |  |  |  |
|  | TA | 0.06 | 6.08 |  |  |  |

**Table S2** Refinement of the composition of the LFP phase in the s-LFP black mass

| atom | x | y | z | Frac. | Uiso. |
| --- | --- | --- | --- | --- | --- |
| Li1 | 0 | 0 | 0 | 0.9588 | 0.03926 |
| Li2 | 0.282 | 0.25 | 0.9738 | 0.0412 | 0.01200 |
| Fe1 | 0.282 | 0.25 | 0.9738 | 0.9588 | 0.00080 |
| Fe2 | 0 | 0 | 0 | 0.0412 | 0.01100 |
| P | 0.0946 | 0.25 | 0.4182 | 1 | 0.01372 |
| O1 | 0.0973 | 0.25 | 0.7394 | 1 | 0.00305 |
| O2 | 0.4548 | 0.25 | 0.2086 | 1 | 0.00874 |
| O3 | 0.1643 | 0.0481 | 0.2843 | 1 | 0.00358 |
| a=10.32198 Å, b=6.00421 Å, c=4.69154 Å, space group: Pnma, 99.5 wt.%LFP, R_wp_: 7.184% | | | | | |

**Table S3** Elemental analysis of the s-LFP black mass

| LFP sample | Li | Fe | | P | Al |
| --- | --- | --- | --- | --- | --- |
| s-LFP | 0.994 | 1 | 0.981 | | 0.0074 |
| LFP-Hydro-TA-HT | 1.011 | 1 | 0.974 | | 0.0066 |

**Table S4** The Rietveld refinement of XRD patterns of relithiated LFP sample at different hydrothermal temperature for different time

| Samples | Phase composition | Fe_Li_ ratio | R_wp_ |
| --- | --- | --- | --- |
| LFP-Hydro-80-10h | 100% LiFePO_4_ | 3.65% | 3.681% |
| LFP-Hydro-95-10h | 100% LiFePO_4_ | 1.16% | 3.426% |
| LFP-Hydro-95-4h | 100% LiFePO_4_ | 2.97% | 3.696% |
| LFP-Hydro-100-4h | 100% LiFePO_4_ | 1.35% | 3.548% |
| LFP-Hydro-120-4h | 100% LiFePO_4_ | 1.14% | 3.534% |
| LFP-Hydro-150-4h | 100% LiFePO_4_ | 0.96% | 3.682% |
| LFP-Hydro-180-4h | 100% LiFePO_4_ | 1.58% | 3.786% |

**Table S5** Refinement of the composition of the LFP phase in the re-LFP

| atom | x | y | z | Frac. | Uiso. |
| --- | --- | --- | --- | --- | --- |
| Li1 | 0 | 0 | 0 | 0.9882 | 0.03926 |
| Li2 | 0.282 | 0.25 | 0.9738 | 0.0118 | 0.01 |
| Fe1 | 0.282 | 0.25 | 0.9738 | 0.9882 | 0.00738 |
| Fe2 | 0 | 0 | 0 | 0.0118 | 0.01 |
| P | 0.0946 | 0.25 | 0.4182 | 1 | 0.01372 |
| O1 | 0.0973 | 0.25 | 0.7394 | 1 | 0.00305 |
| O2 | 0.4548 | 0.25 | 0.2086 | 1 | 0.00874 |
| O3 | 0.1643 | 0.0481 | 0.2843 | 1 | 0.00358 |
| a=10.32198 Å, b=6.00421 Å, c=4.69154 Å, space group: Pnma, R_wp_:3.83% | | | | | |

**Table S6** Comparison of the electrochemical performance of this work with other LFP direct regeneration studies

| Specific capacity mAh g^−1^ | Rate performance | Long-term cycling | Low-temperature performance | Voltage gap (mV) | Ref. |
| --- | --- | --- | --- | --- | --- |
| 170 (0.1 C);  139.1 (1C) | 100.1 mAh g^−1^ at 10 C | 76.6% retention  after 1000 cycles at 1C;  ≈76.8% capacity retention after 1000 cycles at 10 C | 145.7 mAh g^−1^ (25 °C),  82.2 mAh g^−1^ (−20 °C), and 152.8 mAh g^−1^ (50 °C)  0.3C | 58.9  (1 C) | [S1] |
| 146.2 (0.2 C);  141.9 (1 C) | 128.2 mAh g^−1^ at 5 C. | 98.6% retention after 200  cycles at 1 C. | \ | >150  (1 C) | [S2] |
| 145 (0.5 C)  140 (1C) | ~110 mAh g^-1^ at 5C | >95% retention after 100 cycles at 0.5C | \ | >150 | [S3] |
| ~170 (0.1C)  162 (0.2C);  144 (2C); | 102 mAh g^-1^ at 10C | 94.3% retention after 1000 cycles at 0.5 C;  No capacity loss after 300 cycles at 2 C, 5C,10C | \ |  | [S4] |
| ~166 (0.1C);  151.9 (0.5C);  142.07 (1C);  133.11 (2 C) | 114.96 mAh g^-1^ at 5C | 99.1% retention after 200 cycles at 1C;  96.3% retention after 200 cycles at 2 C; 94.7%retention after 200 cycles at 5 C | \ | >150 | [S5] |
| 138.8 (1C);  124.3  (2C) | 107 mAh g^−1^ at 10C | 98.7% retention after 100 cycles at 1C;  87.9% retention after 500 cycles at 1C | \ | \ | [S6] |
| 162.8 (0.2C);  157.4 (0.5C),  147.9 (1C), | 138.4 mAh g^−1^ at 2C;  121.5 mAh g^−1^ at 5C | 99.63% retention after 100 cycles at 0.2C; | \ | \ | [S7] |
| 147.9 (1C),  136.1 (2C) | 113.6, mAh g^−1^ at 5C;  87.2 mAh g^−1^ at 10C | 82% retention after 500 cycles at 1 C rate;  86% after 500 cycles at 5 C. | \ | 83  (0.1 C) | [S8] |
| 167.8 (0.2 C);  141.5 (1 C);  131.6 (2 C) | 109.8 mAh g^-1^ at 109.85C | 92% retention after 300 cycles at 2C | 102.8 mAh g^−1^ at 1.0 C (−5°C). | \ | [S9] |
| ~138 (1C) | 117 mAh g^-1^ at 5C;  97 mAh g^-1^ at 10C | 88% retention after 400 cycles at 5C | ~61 mAh g^-1^ (0.5C, −20°C) | \ | [S10] |
| 140 (1C) | 117 mAh g^-1^ at 5C | 88.5% retention after 400 cycles at 1C | ~61 mAh g^-1^ (0.5C, −20°C) |  | [S11] |
| 135.1 (1C) | 116 mAh g^-1^ at 5C;  105.5 mAh g^-1^ at 10C; | 97.5% retention after 300 cycles at 1C | \ | \ | [S12] |
| 140.1 (1C) | 112.8 mAh g^-1^ at 5C;  93.0 mAh g^-1^ at 10C; | 93.1 % retention after 500 cycles at 5C;  93.8 % retention after 500 cycles at 10C | \ | \ | [S13] |
| 141.3 (1C) | 113.1mAh g^-1^ at 5C;  92.4 mAh g^-1^ at 10C; | 85% after 500 cycles and 72% after 1000 cycles at 1 C | 101.5 mAh g^-1^ (0.5C, 0°C) | 83 mV | [S14] |
| 161 (0.1 C);  151 (0.5C);  146 (1C);  138 (2C) | ~106.5 mAh g^-1^ at 10C  122 mAh g^-1^ at 5C | 69.8% retention (72.3 mAh g^-1^) after 1000 cycles at 10 C ; 67.8% retention （76.37 mAh g^-1^) after 1000 cycles at 8C; 97.5% retention after 100 cycles (5C) | ~62.5 mAh g^-1^ (0.5C, −20°C) | 66 mV  (1C) | This work |

**Text S1** Calculation of D_Li_ using the PITT technique.

The PITT calculation is based on equation (S1)

$\ln\left( i \right)=ln\left( 2\Delta QD_{Li}/L^{2} \right)-\left[ \pi^{2}D_{Li}/{(4L}^{2}) \right]t$ (S1)

For example, the ln(i)-t curve charged to 3.5V was plotted and the linear fit is shown in Figure 3(c). L equals to the electrode depth mines collector depth, which is about 183 μm. k was fitted to be −7.13529×10^−5^

D_Li_ = 7.13529×10^−5^×4×(183×10^−4^)^2^∕π^2^ (cm^2^/s) =9.684×10^−9^ (cm^2^/s)

Based on Equation S1, we calculated the apparent D_Li_ at different voltages during delithiation, as shown in Fig. 3c.

**Supplementary References**

1. K. Jia, J. Ma, J. Wang, Z. Liang, G. Ji et al., Long-life regenerated LiFePO_4_ from spent cathode by elevating the d-band center of Fe. Adv. Mater. **35**(5), 2208034 (2022). <https://doi.org/10.1002/adma.202208034>
2. Q. Jing, J. Zhang, Y. Liu, W. Zhang, Y. Chen et al., Direct regeneration of spent LiFePO_4_ cathode material by a green and efficient one-step hydrothermal method. ACS Sustainable Chem. Eng. **8**(48), 17622-17628 (2020). <https://doi.org/10.1021/acssuschemeng.0c07166>
3. X. Liu, M. Wang, L. Deng, Y.-J. Cheng, J. Gao et al., Direct regeneration of spent lithium iron phosphate via a low-temperature molten salt process coupled with a reductive environment. Ind. Eng. Chem. Res. **61**(11), 3831-3839 (2022). <https://doi.org/10.1021/acs.iecr.1c05034>
4. P. Xu, Q. Dai, H. Gao, H. Liu, M. Zhang et al., Efficient direct recycling of lithium-ion battery cathodes by targeted healing. Joule **4**(12), 2609-2626 (2020). <https://doi.org/10.1016/j.joule.2020.10.008>
5. B. Chen, M. Liu, S. Cao, H. Hu, G. Chen et al., Direct regeneration and performance of spent LiFePO_4_ via a green efficient hydrothermal technique. J. Alloys Compd. **924**, 166487 (2022). <https://doi.org/10.1016/j.jallcom.2022.166487>
6. J. Wang, S. Ji, Q. Han, F. Wang, W. Sha et al., High performance of regenerated LiFePO_4_ from spent cathodes via an in situ coating and heteroatom-doping strategy using amino acids. J. Mater. Chem. A **12**(25), 15311-15320 (2024). <https://doi.org/10.1039/D4TA01098A>
7. W. Song, J. Liu, L. You, S. Wang, Q. Zhou et al., Re-synthesis of nano-structured LiFePO_4_/graphene composite derived from spent lithium-ion battery for booming electric vehicle application. J Power Sources. **419**, 192-202 (2019). <https://doi.org/10.1016/j.jpowsour.2019.02.065>
8. D. Tang, G. Ji, J. Wang, Z. Liang, W. Chen et al., A multifunctional amino acid enables direct recycling of spent LiFePO_4_ cathode material. Adv. Mater. **36**(5), 2309722 (2024). <https://doi.org/10.1002/adma.202309722>
9. Z. Zeng, P. Xu, J. Li, C. Yi, W. Zhao et al., Large-scale and homogenized strategies of spent LiFePO_4_ recycling: Reconstruction of targeted lattice. Adv. Funct. Mater. **34**(6), 2308671 (2024). <https://doi.org/10.1002/adfm.202308671>
10. G. Ji, J. Wang, Z. Liang, K. Jia, J. Ma et al., Direct regeneration of degraded lithium-ion battery cathodes with a multifunctional organic lithium salt. Nat. Commun. **14**(1), 584 (2023). [https://doi.org/10.1038/s41467-023-36197-6](%20https:/doi.org/10.1038/s41467-023-36197-6)
11. J. Li, R. Shi, J. Wang, Y. Cao, H. Ji et al., Interfacial metal-solvent chelation for direct regeneration of LiFePO_4_ cathode black mass. Adv. Mater. **37**(5), 2414235 (2024). <https://doi.org/10.1002/adma.202414235>
12. X. Qiu, C. Wang, Y. Liu, Q. Han, L. Xie et al., Ambient-pressure relithiation of spent LiFePO_4_ using alkaline solutions enables direct regeneration of lithium-ion battery cathodes. J. Energy Storage. **105**, 114721 (2025). <https://doi.org/10.1016/j.est.2024.114721>
13. C. Feng, Y. Cao, L. Song, B. Zhao, Q. Yang et al., Direct regeneration of industrial LiFePO_4_ black mass through a glycerol-enabled granule reconstruction strategy. Angew. Chem. -Int. Ed. **64**(6), e202418198 (2025). [https://doi.org/10.1002/anie.202418198](%20https:/doi.org/10.1002/anie.202418198)
14. Y. Cao, J. Li, D. Tang, F. Zhou, M. Yuan et al., Targeted defect repair and multi-functional interface construction for the direct regeneration of spent LiFePO_4_ cathodes. Adv. Mater. **36**(48), 2414048 (2024). <https://doi.org/10.1002/adma.202414048>
